# Supplementary material for: Influence of C60 Nanofilm on the Expression of Selected Markers of Mesenchymal–Epithelial Transition in Hepatocellular Carcinoma
Source: Cancers (Basel). 2023 Nov 23;15(23):5553. doi: 10.3390/cancers15235553 (PMC10705132; doi:10.3390/cancers15235553)
Supplement: Supplementary file 1 [file cancers-15-05553-s001.zip › cancers-2709985-supplementary.pdf]

Supplementary materials

Influence of C_{60} Nanofilm on the Expression of Selected Markers of Mesenchymal-Epithelial Transition in Hepatocellular Carcinoma

Malwina Sosnowska [1,*], Marta Kutwin [1], Katarzyna Zawadzka [1], Michał Pruchniewski [1], Barbara Strojny [1], Zuzanna Bujalska[1], Mateusz Wierzbicki [1], Sławomir Jaworski [1], and Ewa Sawosz [1]

1 Department of Nanobiotechnology, Institute of Biology, Warsaw University of Life Sciences, Warsaw, Poland; malwina_sosnowska@sggw.edu.pl
* Correspondence: malwina_sosnowska@sggw.edu.pl; Tel.: +48 225936671

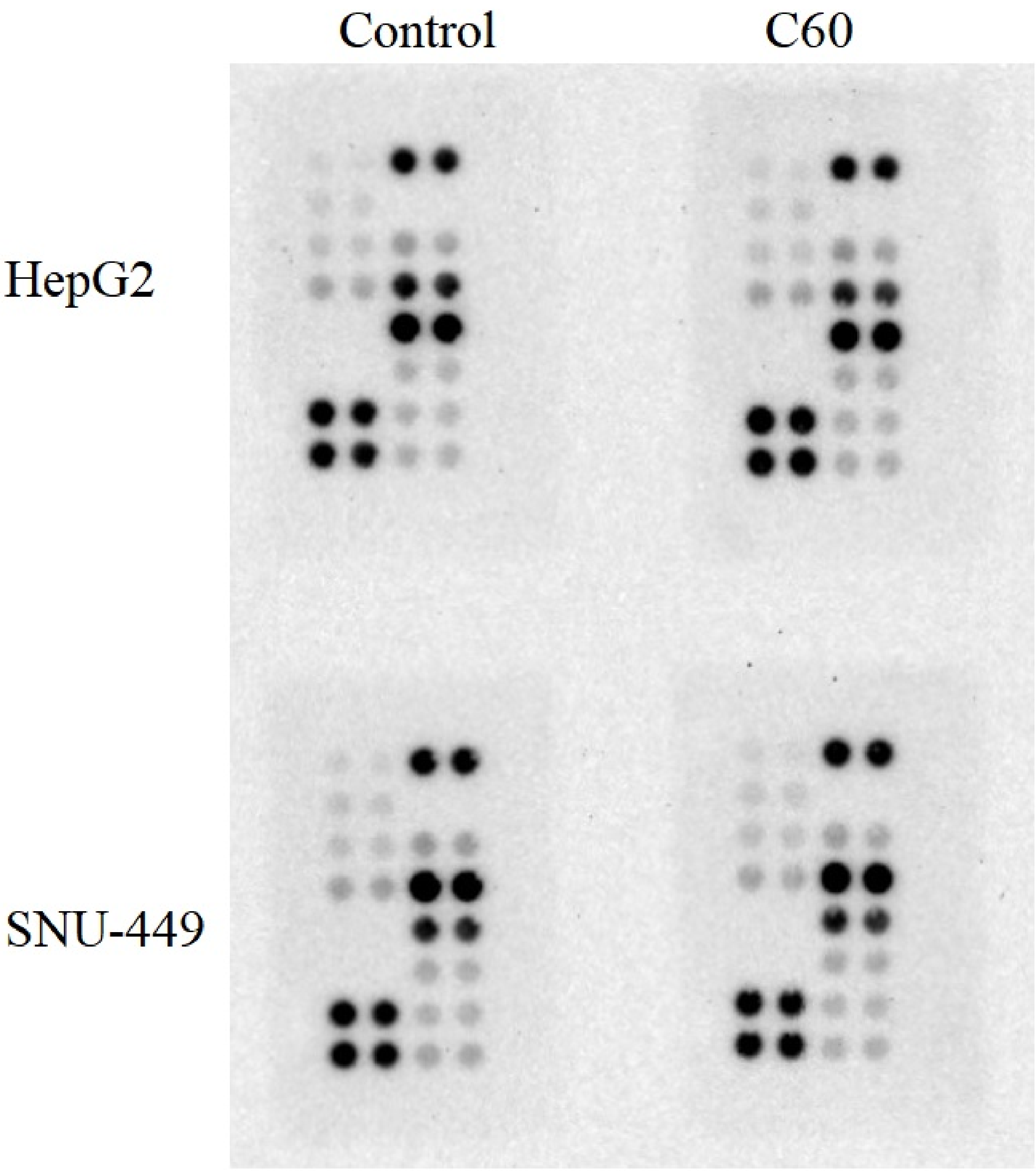

Figure S1. Uncropped antibody arrays of metalloproteinases synthesis and its regulators in HepG2 and SNU-449 cells with or without treatment with fullerene nanofilm.

Table S1. Change in cell area during growth of epithelial or mesenchymal cells on ordinary plate and plate C_{60}-coated plate using ImageJ. Morphometric analysis was performed using 2.5× magnification images from a two-dimensional invasion test (Figure 4). Statistical significance is indicated by asterisks: * $p < 0.05$, ** $p < 0.01$ and *** $p < 0.001$.

Cell line	Phenotype	Research groups	% of cell area	p-value
HepG2	Epithelial	Control	62.45+/-5.46	0.5086
		C_{60}	65.29+/-5.71	
	Mesenchymal	Control	40,38+/-5.60	0.0060
		C_{60}	62.14 +/-2.58**	
SNU-449	Epithelial cells	Control	23.15+/-4.59	0.526
		C_{60}	25.65+/-0.75	
	Mesenchymal	Control	29.95+/-2.62	0.3874
		C_{60}	28.07+/-2.45	
HFF2	Mesenchymal	Control	11.19+/-5.33	0.0585
		C_{60}	18,52+/-3.97	
